# Screening of Natural Product-Derived USP7 Inhibitors for Cancer Therapy via Integrated Machine Learning and Molecular Simulations

**DOI:** 10.3390/cimb48060621

**Published:** 2026-06-16

**Authors:** Faris Alrumaihi

**Affiliations:** Department of Medical Laboratories, College of Applied Medical Sciences, Qassim University, Buraydah 51452, Saudi Arabia; f_alrumaihi@qu.edu.sa

**Keywords:** Deubiquitinases, USP7, small-molecule inhibitors, machine learning, molecular dynamics simulations

## Abstract

Ubiquitination, a crucial cellular protein regulation process, is linked to various diseases, including cancer. Deubiquitinases (DUBs) can reverse ubiquitination, offering a therapeutic strategy. USP7, a DUB, is a key target in oncology due to its role in destabilizing p53, and small-molecule inhibitors could restore p53 activity and combat tumor growth. In this study, we integrated a machine learning (ML)-based screening approach with molecular docking and molecular dynamics (MD) simulations in order to identify potential small-molecule inhibitors of USP7. ML-based screening identified 22 active molecules from a library of 2301 natural compounds. Among the 22 active compounds, only fifteen compounds fulfilled the drug-likeness criteria. Subsequently, molecular docking found three compounds, PubChem 162957515, 114917, and 442879 as potential inhibitors based on binding affinity and interactions. Further, MD simulations and MM-PBSA analyses were performed to evaluate the stability and dynamic behavior of the complexes. Binding energy calculations Molecular Mechanics Poisson–Boltzmann Surface Area (MM-PBSA) revealed that compounds PubChem 114917 and 162957515 exhibited strong binding affinities of −20.98 kcal/mol and −18.68 kcal/mol, respectively, implying that these compounds could serve as promising inhibitors for the development of anticancer therapeutics.

## 1. Introduction

Protein ubiquitination is an important process in eukaryotes that governs a variety of physiological processes, including the removal of damaged or misfolded proteins, cell cycle progression, and signal transmission [1]. Ubiquitination is a process where the small protein ubiquitin is sequentially activated and transferred by enzymes E1, E2, and E3 to attach to substrate proteins, marking them for regulation. This modification controls protein fate, often signaling their degradation via the 26S proteasome [2,3,4,5].

Protein ubiquitination can be altered by the activity of deubiquitinating enzymes (DUBs), which can eliminate ubiquitin by enhancing the breaking of the peptide bond between ubiquitin and protein [6]. The balance between ubiquitination and deubiquitination is tightly controlled; its disruption can cause abnormal cancer pathway activation, faulty protein complex assembly in inflammation, and protein misfolding, all negatively affecting cell function [7]. Approximately 100 human DUBs have been identified and belong mainly to two families: metalloproteases (including JAB1/MPN/Mov34 metalloenzymes (JAMMs) and Zinc finger-containing ubiquitin peptidase 1 (ZUFSPs) and cysteine proteases including ubiquitin C-terminal hydrolases (UCHs), Machado–Joseph disease proteases (MJD), ovarian tumor proteases (OTUs), and ubiquitin-specific peptidases (USPs) [8].

Ubiquitin-specific protease 7 (USP7), a member of the largest USP family of DUBs [9], was first identified in 1997 as a partner of the immediate–early protein and is mainly localized in the nucleus [10]. It plays a crucial role in stabilizing various proteins involved in DNA repair, kinase regulation, transcription, epigenetic gene expression, and viral infections.

Previous studies have reported that several USPs have significant biological functions that make them attractive therapeutic targets [11]. USP7, also known as herpesvirus-associated ubiquitin specific protease, has emerged as a potential oncology target due to its function in regulating the tumor suppressor p53 [12]. Furthermore, it is overexpressed in multiple cancers, such as myeloma [13], prostate [14], glioma [15], hepatocellular carcinoma [16], and ovarian cancer [17]. Additionally, previous studies have explored computer-aided drug discovery approaches targeting downstream pathways such as the p53–MDM2 interaction, underscoring the therapeutic relevance of the ubiquitin system [18,19].

**Figure 1:**
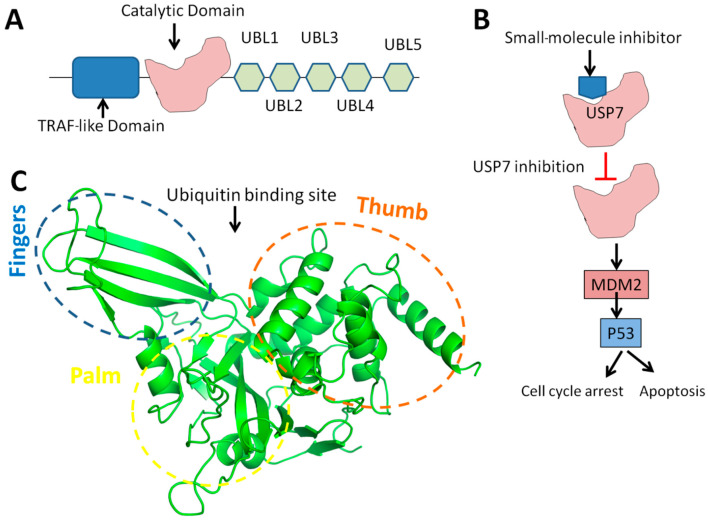
(**A**) Different structural domains of the USP7. (**B**) Schematic of USP7-regulated signaling to combat cancer cells. (**C**) Catalytic core domain structure.

Ubiquitin ligase MDM2 promotes the degradation of the tumor suppressor p53 [20]. USP7 depletion catalyzes the ubiquitination of USP7 substrates, including MDM2. Later, MDM2 proteasomal denaturation stabilizes p53, thus stimulating cell cycle arrest and apoptosis [21]. Upregulation of USP7 also activates cellular signaling pathways like NOTCH and WNT/β-catenin, promoting cancer cell proliferation [22] (Figure 1B). Therefore, inhibition of USP7 enhances the ubiquitination of additional substrates, which modulates the cellular physiology and reactivates the P53 pathways that decrease cancer cell growth [23]. These effects may provide therapeutic benefits in the treatment of multiple cancers.

The USP7 is a 135 kDa protein and comprising seven domains, including the main catalytic domain, the TRAF-like (tumor necrosis factor receptor-associated factor) domain, and five other UBL domains [12] (Figure 1A). The catalytic core is the largest domain of USP7 within the 40 kDa fragment and 208–560 amino acid length. The structure of the catalytic core comprises three subdomains, namely, palm, fingers, and thumb [24] (Figure 1C). Anti-parallel beta-sheet structure forms a deep cleft, and the residues C223, H464, and D481 are located in the cleft between the thumb and palm region [25]. The thiol group of the Cys223 is deprotonated by a histidine 464 residue and triggers a nucleophilic attack on the isopeptide bond, whereas the aspartate causes the histidine to become polarized by restricting its side chain rotation [26]. Several studies have also been reported in the identification of inhibitors against USP7 using different methods such as high-throughput screening (HTS), structure-based drug design (SBDD), virtual screening, and biochemical assay methods [27]. Recently, Kategaya et al. used structure-based studies to identify GNE-6640 and GNE-6776 as small-molecule inhibitors that regulate the enzymatic activity of USP7 by attaching to the unique functional site within the “palm” location of the enzyme to prevent cancer [28]. Traditional drug discovery approaches, while foundational, exhibit limitations in their scope and capacity to uncover novel compounds. Over the past decade, AI models have greatly revolutionized the field of drug discovery [29]. Given the limitations of single-method pipelines in virtual screening, a hybrid machine learning (ML) and computer-aided drug discovery (CADD) approach was employed in this study. This hybrid approach offers a significant advantage over conventional drug discovery methods.

The discovery of small-molecule inhibitors by targeting a specific protein is the trending method of drug discovery [30,31,32,33,34,35]. Innovations in supercomputing, algorithms, and computational tools have further enhanced the efficiency of lead compound identification in pharmaceutical research [36,37,38,39,40,41,42]. Integrating machine learning (ML)-based virtual screening with molecular dynamics simulations offers a promising approach for identifying potential inhibitors targeting the catalytic core domain of USP7 in cancer, providing new avenues for combating this prevalent disease. In the current study, ML-based screening integrated with molecular dynamics simulations was employed to identify potent inhibitor candidates for USP7. A library of phytochemicals was then screened, prioritizing those with strong drug-like properties and the potential to inhibit USP7 activity.

## 2. Methods

### 2.1. Data Processing and Features Calculation

Inhibitors reported to exhibit inhibitory activity against *USP7* were retrieved from the BindingDB (https://www.bindingdb.org/rwd/bind/index.jsp, accessed on 6 February 2026) database without applying additional selection criteria. All compounds annotated in BindingDB as inhibitors of USP7 were included in the dataset. BindingDB assigns the inhibitor label to a compound based on reported experimental activity values such as IC_50_ or K_i_, and we adopted this classification directly without imposing further activity cutoffs. These compounds were chosen for their relevance to the protein target USP7 and their experimentally determined inhibitory activity. The reported activity values of the retrieved USP7 inhibitors ranged from 0.16 to 2,000,000 nM. Prior to feature calculation, duplicate inhibitors were removed from the dataset. Subsequently, a total of 30 molecular descriptors were calculated to quantitatively represent the structural and physicochemical properties of each compound [43,44,45]. These features were selected to capture key aspects such as molecular size, lipophilicity, electronic distribution, topological complexity, and pharmacophoric characteristics, which are important for biological activity prediction using the RDKit package version 2019.09.1 [46]. Additionally, 2000 decoy molecules were generated using the Directory of Useful Decoys-Enhanced (DUD-E) (https://dude.docking.org, accessed on 6 February 2026) database [47]. The active inhibitors from the BindingDB database were tagged as 1, denoting their status as actives. The generated decoy molecules were designated 0 to indicate their expected inactivity against the protein target. All active inhibitors and decoy compounds, together with their labels, were combined into a single dataset. The chemical structures of the active inhibitors and decoy compounds were represented using the SMILES (Simplified Molecular Input Line Entry System) notation. The dataset was stored as an Excel file for easy access and analysis. To address class imbalance and improve model generalization, the Synthetic Minority Oversampling Technique (SMOTE) was applied to the training dataset. SMOTE generates synthetic samples of the minority class, thereby balancing the class distribution and reducing prediction bias toward the majority class (Figure S1). The dataset was then divided into training and testing subsets, with 70% for training and 30% for testing.

### 2.2. Principal Component Analysis (PCA)

Principal component analysis (PCA), a popular dimensionality reduction approach, was employed to streamline the dataset by its variability into a smaller number of features [48]. PCA works by converting the original set of correlated variables into a new set of uncorrelated variables called principal components, which effectively encapsulate the data’s most significant patterns. To do this, the Scikit-learn library [49] was used to generate a PCA model with two principal components. The model was then fitted to the dataset’s selected characteristics, and the resulting main components were used for further analysis and processing. This method not only decreased computing complexity but also boosted the interpretability of the data by focusing on its most important aspects.

### 2.3. Machine Learning Models and Evaluation

Various machine learning models were constructed and thoroughly tested to determine if the compounds in the dataset were active or inactive. These models were built using the preprocessed dataset, which ensured that the data was fit for rigorous analysis. The algorithms implemented in this study included Support Vector Machine (SVM) [50], K-Nearest Neighbors (KNN) [51], Naive Bayes (NB) [52], and Random Forest [53], each of which has unique strengths and approaches for classification tasks. Hyperparameter optimization was performed using GridSearchCV to identify the optimal parameter combinations for each machine learning algorithm. The tuning process was conducted on the training dataset to improve predictive performance and reduce the risk of model overfitting. To confirm the credibility of the results, we used a 10-fold cross-validation procedure. Each machine learning model’s performance was evaluated using a variety of statistical criteria, including accuracy, sensitivity, specificity, and the Matthews Correlation Coefficient. In addition, precision, F1-score, and the area under the receiver operating characteristic curve (ROC-AUC) were calculated to provide a comprehensive assessment of classification performance. Accuracy provides an overall measure of the models’ correct predictions, while sensitivity and specificity measure the models’ ability to correctly identify active and inactive compounds, respectively. This detailed evaluation not only helps to identify the most effective model for distinguishing active compounds from inactive ones but also highlights areas for potential improvement.

### 2.4. Machine Learning Model-Based Screening

An MPD3 library [54] consisting of 2301 natural compounds was retrieved, to find potential bioactive compounds against USP7. Phytochemicals were chosen over synthetic compounds due to their structural diversity, biocompatibility, and lower toxicity, which make them promising candidates for drug discovery and development particularly valuable in complex diseases like cancer [55,56]. This dataset was rigorously processed, and the feature computation procedures that we built for the above dataset were methodically applied to this new target dataset. Once the dataset was processed, the pre-trained machine learning model was employed to classify each compound in the library as either active or inactive. The pre-trained model facilitated an efficient and high-throughput screening method, utilizing its predictive capacity to discover compounds with potential biological activity. This method dramatically sped up the discovery process by choosing good candidates for further study. After screening, the compounds identified as active were further screened based on Lipinski’s rule of five [57], a widely accepted set of criteria for evaluating drug-like properties. This rule evaluates essential physicochemical parameters such as molecular weight, lipophilicity (logP), hydrogen bond donors, and hydrogen bond acceptors to predict a compound’s ability to be orally active in humans. Using these rule of five criteria, the pool of active compounds was further narrowed to exclude those that were unlikely to have acceptable pharmacokinetics or bioavailability.

### 2.5. Molecular Docking Protocol

The catalytic domain structure of USP7 complexed with the inhibitor GNE6776 was downloaded from the Protein Data Bank (PDB) (PDB ID 5UQX) (https://www.rcsb.org/structure/5UQX, accessed on 6 February 2026). Protein structure was preprocessed and refined before proceeding with molecular docking. This involved removing other crystallographic components, including water molecules and other non-relevant small-molecules. Following this, polar hydrogens and charges were added to the protein using AutoDockTools version 1.5.6 [58]. For molecular docking experiments Autodock Vina [59] was utilized because it is efficient and reliable in predicting binding interactions. The docking grid box was precisely positioned on the USP7 ubiquitin binding site. The grid box dimensions were set to X = 35 Å, Y = 35 Å, and Z = 35 Å to cover the binding site and allow ligands to explore binding orientations. The exhaustiveness value was set to 16 and twenty binding modes were generated for each ligand, allowing for a thorough evaluation of possible interactions. The predictive ability of the docking protocol was tested by redocking the crystallized ligand GNE6776 into the binding of the USP7.

### 2.6. Molecular Dynamics (MD) Simulation

MD simulations were performed using GROMACS 2024 [60] to thoroughly examine the binding stability and dynamic behavior of ligand–protein complexes in solvent environment. The charmm36-jul2022 force field [61] was utilized to determine protein topology. The ligands were first geometry optimized using Avogadro 1.20 and MOPAC 22.1.1 semi-empirical method. This step helped refine the 3D conformation and remove any initial strain or unrealistic geometry. Following this, ligand topology and force field parameters were generated using the CGenFF web server [62]. The ligand–protein complexes obtained from docking were solvated in a cubic simulation box using the TIP3P water model with dimensions of 8.32276 × 8.32276 × 8.32276 nm^3^, ensuring a minimum distance of 1.0 nm between the complex and the box edge [63]. The system was solvated using the TIP3P water model and 0.15 M NaCl was added to mimic physiological ionic strength and to neutralize the system charge. Energy minimization was performed with 1500 steps of the steepest descent method to optimize the system’s configuration with a maximum force convergence criterion of <1000 kJ/mol/nm, to relieve steric clashes and optimize initial geometry. Subsequently, equilibration was carried out in two phases. First, under the NVT ensemble, the system temperature was gradually increased from 0 K to 300 K over 300 ps, and then under the NPT ensemble, equilibration was maintained at 1.0 bar pressure and 300 K for another 300 ps. The Particle Mesh Ewald (PME) method was utilized for long-range electrostatic interactions. To enable efficient simulations, the LINCS (LINear Constraint Solver) algorithm [64] was employed to constrain all bonds. A 2 fs time step was used with the Verlet cutoff scheme. Production runs were conducted for 100 ns for each complex. Two independent 100 ns simulations were conducted for each system to improve the robustness and reliability of the results. Performing separate runs helps ensure that the observed behaviors are reproducible and not artifacts of a single simulation trajectory. The 100 ns time was chosen based on the previous research which found that simulations of this time were sufficient to monitor the stabilization of ligand–protein interactions [65,66,67]. Post-simulation analyses were performed using GROMACS modules including RMSD, RMSF, SASA, radius of gyration, and PCA.

### 2.7. Binding Free Energy Calculation

The MM/PBSA (molecular mechanics/Poisson–Boltzmann surface area) approach was used to calculate the binding free energies of the USP7–ligand complexes, providing quantitative insights into the stability of these complexes [68]. The Gmx_MMPBSA module [69] was used to perform these calculations, offering an efficient and accurate framework for estimating the binding energies of the simulated complexes. To ensure robust and representative energy calculations, binding free energy snapshots of last 25 ns were extracted at regular intervals of 5 ps from the 100 ns molecular dynamics simulation. The binding free energies in solution were calculated using the following equation:

(1) ΔGbind=ΔGcomplex−[ΔGreceptor+ΔGligand]

In Equation (1) each energy term is calculated as follows:

(2) $${\Delta G}_{binding}={\Delta E}_{MM}+{\Delta E}_{solvation}-T\Delta S$$

${\Delta E}_{MM}$ corresponds to molecular mechanics energy. ${\Delta E}_{MM}$ includes non-bonded energy (electrostatics and van der Waals interaction energy) and bonded energy.

(3) $${\Delta E}_{MM}={non\text{-}bonded\;energy(\Delta E}_{elec}+{\Delta E}_{vdw})+bonded\;energy$$

${\Delta E}_{solvation}$ is solvation free energy, includes polar solvation (ΔEGB) and non-polar solvation energy (ΔESURF). The term −TΔS in Equation (1) represents the entropic contribution to the total binding free energy. In MM-PBSA calculations, this entropic term is excluded, and the resulting value reflects the effective binding free energy. This approximation is generally sufficient when comparing the relative binding affinities of structurally similar ligands. Given the computational cost and potential inaccuracies associated with entropy estimation, it is often omitted when the primary goal is a relative comparison rather than absolute free energy determination [69].

## 3. Results

### 3.1. Data Retrieval and Processing

The dataset of 965 known-to-be-active inhibitors against USP7 was obtained from BindingDB, and 2872 decoy compounds were generated using DUD-E. The active compounds in the dataset retrieved from Binding DB were labeled as 1, and inactive compounds (decoys) were labeled 0 (File S1). The integrated dataset consisting of 3837 compounds was split into training and test sets consisting 2686 (70%) and 1151 (30%) compounds, respectively (Files S2 and S3). Features were calculated of the datasets using RDKit library. The names and statistics of the calculate features are shown in Table 1.

### 3.2. Principal Component Analysis Based Machine Learning Feature Reduction

**Figure 2:**
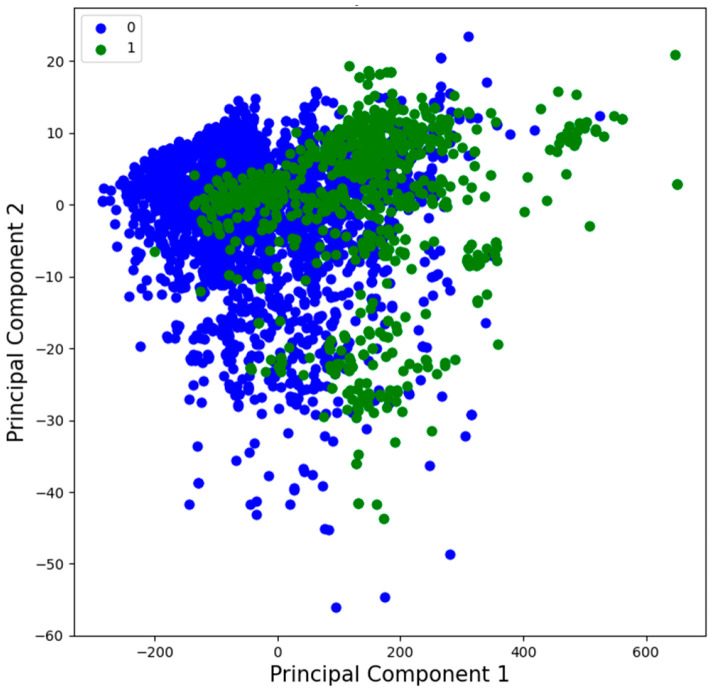
Scatter plot of PC1 vs. PC2 showing separation of active and inactive compounds.

Principal component analysis (PCA) was conducted on the dataset to reduce its dimensionality and extract the most significant patterns in the data. The analysis revealed that the first principal component (PC1) had an eigenvalue of 19,783.74, while the second principal component (PC2) had an eigenvalue of 97.32. These eigenvalues represent the amount of variance captured by each component, with PC1 accounting for an overwhelming 99.44% of the total variance, and PC2 contributing only 0.56%. This indicates that nearly all of the meaningful information in the original high-dimensional data is captured by PC1, while PC2 captures a relatively minor portion, possibly reflecting noise or subtle secondary patterns. The scatter plot (Figure 2) of the transformed data in the PC1–PC2 space illustrates how the dataset is distributed along these two principal components. The blue and green points, representing two different classes (0 and 1), show some degree of separation in this reduced-dimensional space. PC1, being the dominant component, captures the most significant variation, suggesting that the primary structure or key patterns in the data align along this axis. Meanwhile, PC2, though much less significant in terms of variance contribution, may highlight nuanced or orthogonal features that add slight differentiation to the dataset. The results demonstrate the efficiency of PCA in reducing dimensionality while retaining most of the dataset’s critical information. By focusing on PC1 alone, a significant reduction in complexity can be achieved without a substantial loss of information. The inclusion of PC2 adds a minimal layer of additional detail but does not significantly enhance the explained variance. These findings can be used to inform downstream tasks such as visualization, clustering, or machine learning, where reducing dimensionality often improves computational efficiency and model performance. These PCA results informed the feature selection process for subsequent machine learning model training by effectively reducing the dimensionality of the data while preserving key patterns. This reduction not only simplifies the dataset but also improves computational efficiency and can enhance the predictive performance of the models in downstream analysis.

### 3.3. Model Evaluation

**Figure 3:**
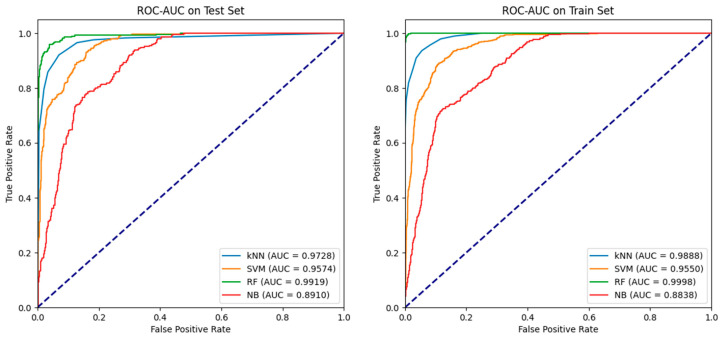
The ROC-AUC of the machine learning models on the test set.

**Figure 4:**
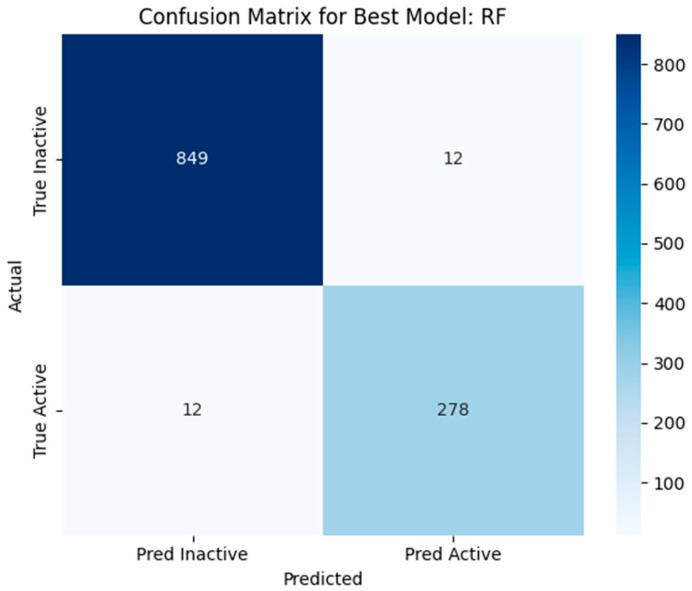
Confusion matrix showing the classification results.

Several supervised machine learning models were developed and assessed using a range of performance metrics, including accuracy, sensitivity, specificity, Matthews Correlation Coefficient (MCC), and area under the receiver operating characteristic curve (AUC). The Random Forest (RF) method outperformed the other models, with an excellent accuracy of 96% and an MCC of 0.90. Following RF, the KNN model performed well, with an accuracy of 93% and an MCC of 0.82. The SVM model stands at third with an accuracy of 85% and an MCC of 0.69. In contrast, the Naïve Bayes model showed the lowest performance, achieving an accuracy of 80% and an MCC of 0.55. A detailed comparison of these metrics for all models is presented in Table 2. To guarantee the reliability and robustness, a 10-fold cross-validation procedure was used. This strategy reduces the chances of overfitting and offers a more accurate evaluation of model performance across diverse data subsets. Furthermore, the AUC of the receiver operating characteristic (ROC) was utilized as a reliable metric to assess the models’ discriminating capacity. The RF model once again demonstrated superior performance, achieving an AUC of 0.99. Figure 3 shows the contrasting AUC performances. The RF model correctly classified 849 true negatives and 278 true positives, with only 12 false positives and 12 false negatives. The test set used for final evaluation comprised 1151 compounds (861 inactive and 290 active), as shown in the confusion matrix (Figure 4). This resulted in a high accuracy of 98%, sensitivity of 95.8%, and specificity of 98.6%, indicating strong predictive performance. The balanced distribution of errors further supports the model’s robustness and reliability for classification tasks.

### 3.4. ML Model-Based Screening on the New Dataset and Applicability Domain (AD) Analysis

The Random Forest (RF) model outperformed other machine learning models, especially in terms of accuracy and the Matthews Correlation Coefficient (MCC). The strong performance of the RF model is further underscored by its high area under the curve (AUC) value, which indicates its excellent ability to distinguish between classes. Among all evaluated models, the RF model achieved the highest AUC. Due to its outstanding performance, the RF model was selected for further application in classifying actives in a library of 2301 natural compounds against USP7. Out of 2301 compounds, 22 were predicted to exhibit activity against USP7 protein (File S4). Among these, three duplicate compounds were identified and removed, resulting in a final set of 19 active compounds. To refine the selection of potential candidates, key drug-like properties of the 19 active compounds were evaluated using the Lipinski’s rule of five. Among the 19 active phytochemicals, 15 met the criteria outlined by Lipinski’s rule, suggesting their strong potential for further exploration as drug candidates (Supplementary file (File S5.csv)).

Additionally, to assess the reliability and robustness of the ML predictions, an applicability domain (AD) analysis was performed for the 19 selected active compounds. The AD was evaluated using a PCA-based chemical space approach, where the training dataset was used to define the model’s descriptor space after standardization of molecular descriptors. The same PCA transformation was then applied to the predicted active compounds to ensure consistency in chemical space representation. This approach was employed to determine whether the predicted compounds fall within the chemical space covered by the training data, which is essential for evaluating the reliability of ML-based predictions. The results indicated that, out of 19 active compounds, 17 compounds were located within the applicability domain, while 2 compounds fell outside the defined chemical space boundary (Figure S2). This suggests that the majority of predicted actives lie within the reliable prediction space of the model, thereby supporting the robustness of the ML model screening approach.

### 3.5. Molecular Docking Results

**Figure 5:**
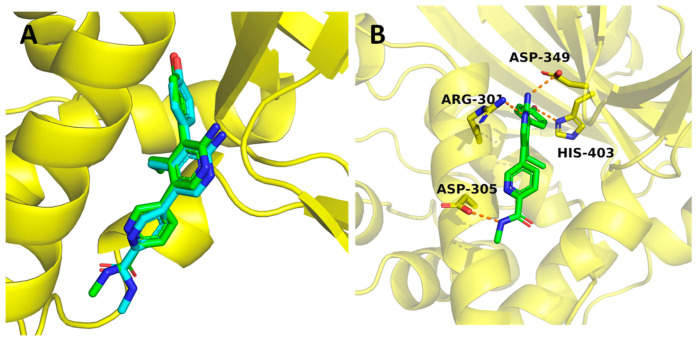
(**A**,**B**) Docking protocol validation. (**A**) Redocked conformation of the native ligand GNE6776 (blue) superimposed to crystallographic conformation green and the protein is in yellow color, (**B**) Interctaion profile of the native ligand made with USP7.

After machine learning model-based screening, the screened compounds predicted to be active against target protein USP7 were docked into the ubiquitin binding site of USP7 in order to found their binding interactions and conformation with the USP7. In order to validate the docking protocol, the native ligand GNE6776 was redocked into the ubiquitin binding site (Figure 5A). The native ligand was extracted from the binding pocket, and then redocked using the same docking parameters used for subsequent screening. The accuracy of the docking was evaluated by comparing the redocked pose with the experimental binding conformation. The RMSD valued for the redocked ligand superimposed to co-crystallized ligand was 1.1 Å, which is within the widely accepted threshold for accurate pose reproduction in docking studies [70,71]. The native ligand formed interactions with the residues Arg301, Asp305, Asp349, and His403 with binding energy score −9.9 kcal/mol.

**Figure 6:**
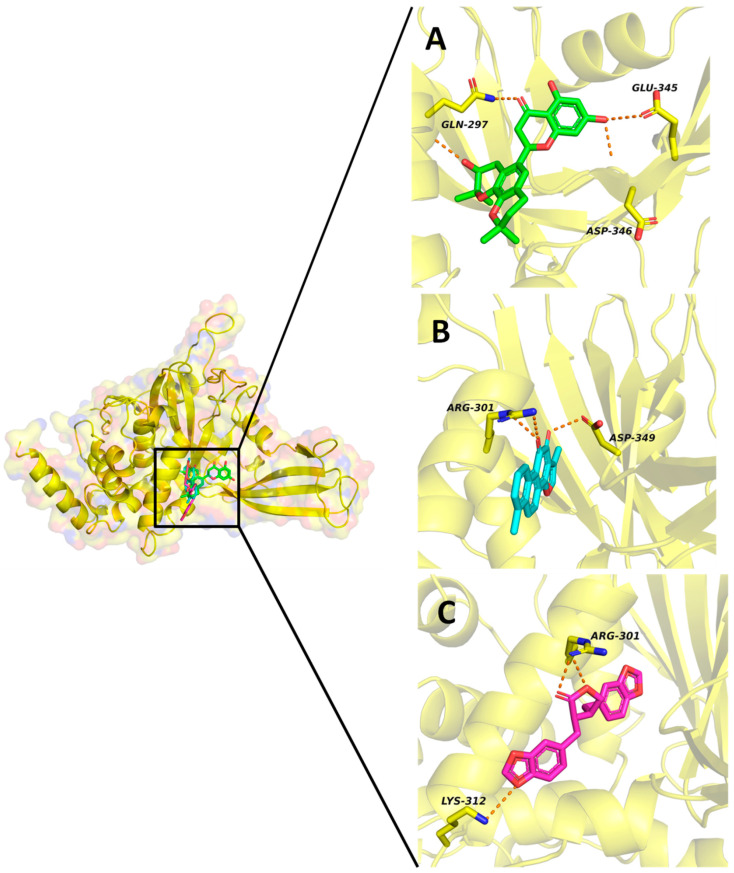
Molecular docking interactions of selected compounds with USP7. The left panel shows the overall binding pose of the ligand within the protein binding pocket, where the protein is represented in yellow cartoon and surface representation, and the ligand is highlighted in stick representation within the active site. The right panels (**A**–**C**) show detailed 2D interaction views of the docked complexes. (**A**) PubChem 162957515, (**B**) PubChem 114917, (**C**) PubChem 442879.

After molecular docking, the compounds were ranked based on their binding affinity scores (kcal/mol). The binding scores of the 15 docked compounds are presented in Supplementary Table S1. Docked conformations of the compounds were then visualized using PyMol 3.1 (https://www.pymol.org/). Three compounds, PubChem 162957515, 114917, and 442879, having interactions with the crucial active site residue and occupying the similar binding position to the native ligand were selected. The binding affinity of these compounds was higher than the native ligand score. The docking results illustrated in Figure 6, and Table 3, demonstrate the binding interactions between the selected ligands and the USP7. All the compounds formed several hydrogen bonds. The proximity of the ligand to these residues suggests a strong interaction, enhancing its potential as an inhibitor. The presence of multiple hydrogen bonds between the ligand and the protein backbone enhances the binding affinity, which contributes to the ligand’s effectiveness as a competitive inhibitor.

The docking results (Table 4) showed that PubChem 162957515 had the highest binding affinity to the USP7 catalytic site, with a binding energy of −11.3 kcal/mol. This compound made four hydrogen bonds with residues Glu345, Asp346, and Gln297. Specifically, the side chain carboxylate oxygen atom of Glu345 and the backbone carbonyl oxygen atom of Asp346 were involved in hydrogen bond interactions with the ligand. In addition, the side chain amide nitrogen atom of Gln297 helped to form the hydrogen bonding network. PubChem 114917 had a binding energy of −10.6 kcal/mol and established three hydrogen bonds with the residues Arg301 and Asp349. The side chain nitrogens of Arg301 and the side chain carboxylate oxygen atom of Asp349 formed hydrogen bonds with the ligand. Similarly, PubChem 442879 had a binding energy of −10.2 kcal/mol and formed three hydrogen bonds. These interactions occurred between the side chain nitrogens of Arg301 and the side chain amino group of Lys312. Arg301 and Gln297 act as a key anchoring residue within the allosteric pocket, stabilizing ligand binding. Glu345 and Asp346 lie near the flexible loop regions and contribute to ligand positioning through electrostatic interactions.

The reference ligand, GNE6776, had a slightly lower binding affinity (−9.9 kcal/mol) but generated four hydrogen bonds (Figure 5B). These bonds involved the side chain nitrogen group of Arg301, the backbone carbonyl oxygen atom of Asp305, the side chain carboxylate oxygen atom of Asp349, and the side chain imidazole nitrogen atom of His403. Collectively, these interactions highlight the importance of both side chain and backbone atoms of key residues in stabilizing the ligand within the USP7 catalytic site.

### 3.6. PASS Online Analysis of Candidate Compounds Against USP7

To evaluate the potential anticancer activities of the screened compounds, PASS (Prediction of Activity Spectra for Substances) analysis was conducted. This tool predicts various biological activities in the human body by calculating the probable activity (Pa) and probable inactivity (Pi) for each compound. The activities relating to Pa > Pi values are presented in Table 5. These results indicate a range of anticancer and antineoplastic activities against different cancers. All three compounds demonstrated potential anticancer activities, as evidenced by Pa values greater than 0.2 across multiple cancer types. Each compound demonstrated predicted overall antineoplastic activity, along with specific indications against breast cancer, cervical cancer, sarcoma, and brain cancer. While the Pa values differed between compounds, all had overlapping activity patterns, indicating broad-spectrum anticancer potential. These results support the fact that the selected compounds could be effective against a wide range of cancer types. Their different anticipated activity patterns indicate that they are promising leads for further in vitro and in vivo validation in anticancer medication development.

### 3.7. Molecular Dynamics (MD) Simulations

**Figure 7:**
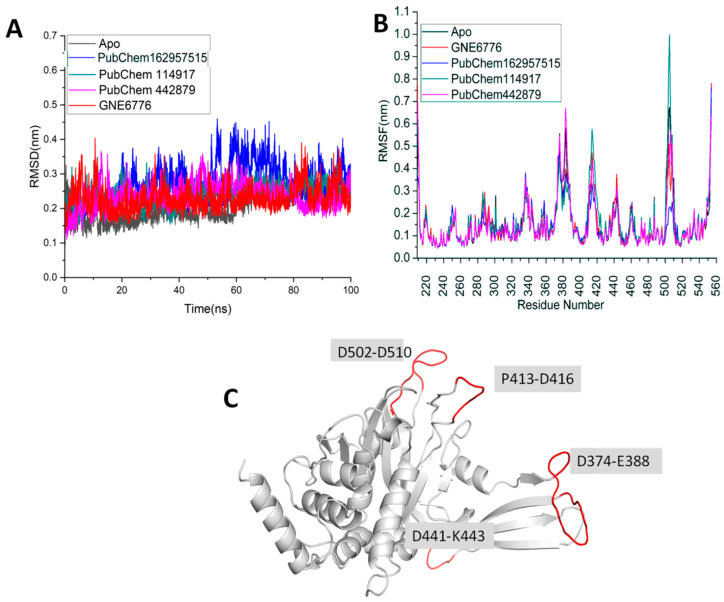
(**A**) RMSD and (**B**) RMSF of the Apo, reference GNE6776, and candidate ligands. (**C**) The loop regions of USP7 with high flexibility are highlighted in red color.

RMSD analysis demonstrates the overall structural stability of ligand–protein complexes during molecular dynamics simulations. RMSD measures the average deviation of the protein or ligand structure over time from its initial conformation. Figure 7A depicts dynamic stability of the simulated systems across the simulation trajectory, indicating how well the systems remained stable (Table 6). Overall, all complexes had modest RMSD values, indicating robust binding interactions throughout the simulations. Among all the systems, PubChem 114917 and 442879 showed the little variations, indicating their strong structural stability. Notably, compound PubChem 442879 had the lowest average RMSD value of 0.20 nm, demonstrating its stability throughout the simulation time. In comparison, the reference compound GNE6776 has an RMSD of 0.23 nm. On the other side, the PubChem 162957515 complex had the greatest RMSD value of 0.27 nm. This increased deviation indicates more conformational flexibility and lower structural stability as compared to GNE6776 and other ligand–protein complexes. These results indicate that PubChem 162957515 may have weaker binding interactions or a more flexible binding mode inside the protein’s active region.

The RMSF (Root Mean Square Fluctuation) analysis of ligand–protein complexes reveals information on the flexibility of individual residues during molecular dynamics simulations. It helps identify regions of the protein that are highly flexible or rigid, often indicating functionally important areas. All the simulated systems showed residual fluctuations below 0.3nm indication stability of the systems. Figure 7B depicts changes in residue locations for the Apo structure, GNE6776, and the selected compounds PubChem 162957515, 114917, and 442879. Notably, the loop areas D374-E388, P413-D416, D502-D510, and D441-K443 had RMSF values greater than 0.3 nm, indicating more flexibility in these areas. These regions, likely corresponding to mobile loops, may play a role in binding interactions or structural adjustments during the simulation. The higher fluctuations in these specific regions are consistent across the different systems, as highlighted in the RMSF graph. These loop regions are further emphasized in the structural representation of USP7 in Figure 7C.

### 3.8. Solvent-Accessible Surface Area (SASA) and Compactness

**Figure 8:**
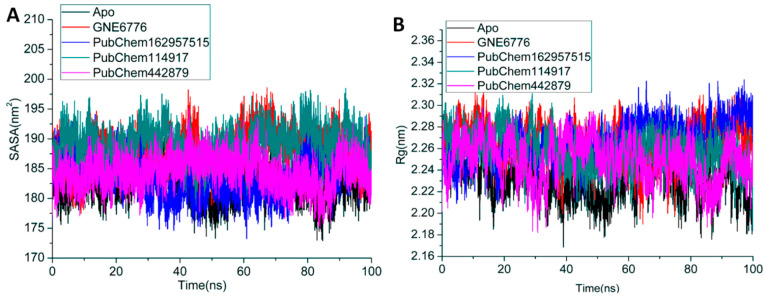
(**A**,**B**) Solvent-accessible surface area and radius of gyration of all the simulated complexes plotted over the time of 100 ns.

SASA measures the surface area of a molecule that is accessible to solvent molecules. It is a significant characteristic for determining molecular compactness and solvent interactions. It provides insight into protein folding, and how buried or exposed the ligand or binding site becomes over time. Among the simulated systems, PubChem 442879 and 162957515 had the lowest SASA values (Figure 8A), indicating the most compact structural conformations with little solvent exposure. This suggests that these complexes have tightly packed structures, which might be caused by particular ligand interactions or increased molecular stability. On the other side, PubChem 114917 has the greatest SASA value, indicating a more open and solvent-accessible structure. This higher SASA suggests a less compact conformation, which may allow for more solvent interaction and flexibility in its molecular architecture. The observed SASA differences across different systems highlight the impact of ligand binding and molecular interactions on structural dynamics and solvent accessibility. These findings emphasize the importance of individual ligands in regulating molecular compactness and the overall solvent interaction landscape of the systems.

The radius of gyration (Rg) is an important measure for determining the compactness and structural stability of molecular systems. A relatively stable Rg profile throughout the simulation indicates that the structural integrity of the protein is maintained during the trajectory. Rg of all the simulated systems is demonstrated in Figure 8B. Variations in Rg values offer information on the structural dynamics affected by ligand binding. Among the simulated systems, Pubchem 442879 and 114917 had the lowest Rg values, indicating strong intramolecular interactions and a very compact shape. PubChem 162957515 and PubChem 114917 exhibit nearly similar Rg profiles, closely comparable to the reference compound GNE6776. This suggests that both ligands maintain a comparable level of structural stability and compactness in the protein system without inducing major conformational changes. The comparable Rg behavior of PubChem 162957515 and PubChem 114917 with the reference compound GNE6776 confirms their stable binding profiles and similar structural impact on the protein system. These changes may indicate a balance of stability and flexibility, allowing these complexes to evolve while retaining structural integrity. Overall, the Rg results demonstrate how various ligands affect the compactness and structural structure of the USP7 system, highlighting their influence on protein stability throughout the simulation.

### 3.9. Post-Simulation Interaction Analysis

**Figure 9:**
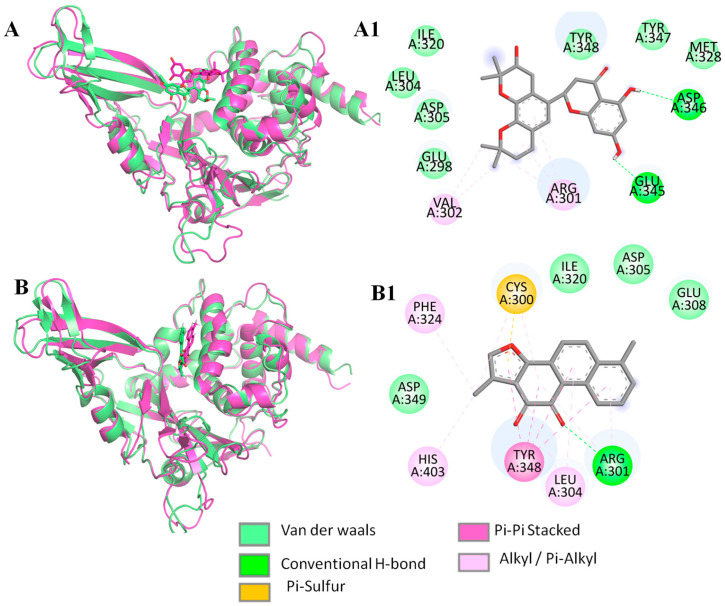
(**A**) Post-simulation ligand–protein complex stability of PubChem 162957515. Complex extracted at 0 ns (Green) is superimposed to complex extracted at 100 ns (Pink) and (**A1**). The interaction between PubChem 162957515 and the protein at 100 ns time. (**B**) Post-simulation ligand–protein complex stability of PubChem 114917. (**B1**) The interaction between PubChem 114917 and the protein at 100 ns time.

Post-simulation interaction analysis was performed to assess the stability and binding interactions of the top-scoring compounds, PubChem 162957515, PubChem 114917, and PubChem 442879 with USP7. Out of the three compounds, PubChem 442879 moved away from the active site of the USP7, while PubChem 162957515 and PubChem 114917 remained stably bound inside the pocket of USP7. The structural superimposition of ligand–protein complexes at 0 ns and 100 ns stable binding over the course of the 100 ns simulation is shown in Figure 9A,B for PubChem 162957515 and PubChem 114917, respectively. PubChem 162957515 maintained consistent interactions with key active site residues, including ARG301, ASP305, GLU298, GLU345, and TYR348, suggesting a strong and stable binding conformation within the catalytic pocket (Figure 9A1). Similarly, PubChem 114917 also exhibited stable binding with USP7 throughout the simulation (Figure 9B1). At 100 ns, it formed critical interactions with residues such as CYS300, ARG301, TYR348, and ASP305. Overall, both compounds retained stable conformations and crucial interactions during the simulation period, supporting their potential as promising USP7 inhibitors.

### 3.10. Hydrogen Bond Analysis

**Figure 10:**
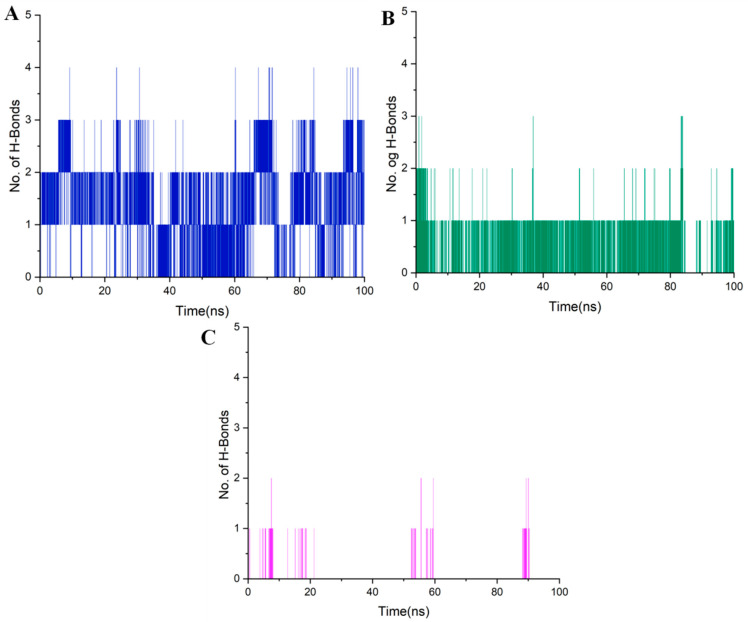
Number of hydrogen bonds formed between ligand and protein over the time of 100 ns simulation. (**A**) PubChem 162957515, (**B**) PubChem 114917, (**C**) PubChem 442879.

Hydrogen bond analysis was performed to evaluate the stability and strength of interactions between each ligand and the target protein over the course of a 100 ns simulation. PubChem 162957515 (Figure 10A) consistently formed 1 to 3 hydrogen bonds throughout the simulation, with occasional spikes reaching up to 4, indicating a stable and persistent interaction with the protein binding pocket. This suggests that PubChem 162957515 maintained a strong binding orientation and contributed to the structural integrity of the complex. In contrast, PubChem 114917 (Figure 10B) formed 1 to 2 hydrogen bonds with the protein. Meanwhile, PubChem 442879 (Figure 10C) showed minimal hydrogen bond formation, with infrequent and short-lived interactions mostly limited to single bond. This pattern indicates poor binding stability and suggests that 442879 may not effectively anchor within the active site. As mentioned above, this ligand detached from the protein after some time.

### 3.11. Post Simulation Principal Component Analysis

**Figure 11:**
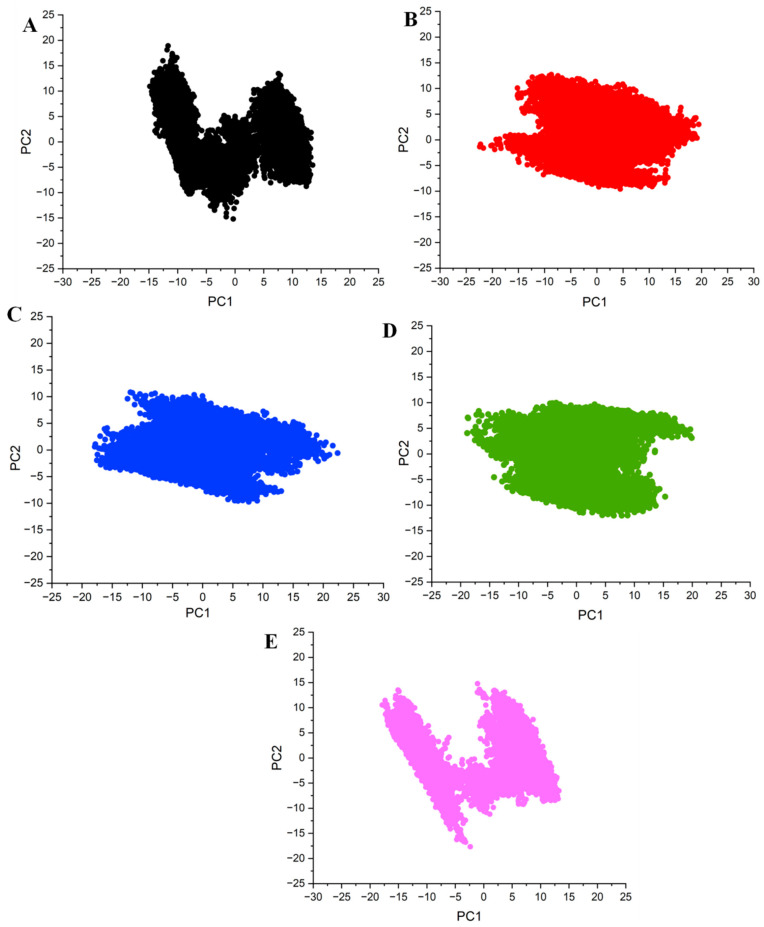
Principal component analysis of (**A**) apo-USP, (**B**) Reference-GNE6776, (**C**) PubChem 162957515, (**D**) PubChem 114917 (**E**) PubChem 442879.

Principal component analysis (PCA) was employed to explore the collective motions and conformational stability of the protein–ligand complexes during the molecular dynamics simulations. The PCA plots represent the projection of the trajectories on the first two principal components (PC1 and PC2), highlighting the conformational space sampled by each system. As shown in Figure 11A, the apo-USP system exhibited a wider and more scattered distribution, indicating greater conformational flexibility in the absence of any ligand. In contrast, the reference complex with GNE6776 (Figure 11B) showed a more compact and confined motion, suggesting that ligand binding stabilizes the protein structure. Similarly, PubChem 162957515 (Figure 11C) displayed a dense and localized cluster, reflecting restricted motion and structural stability upon binding. PubChem 114917 (Figure 11D) and PubChem 442879 (Figure 11E) also showed relatively compact distributions, though with slightly broader scattering compared to the reference and 162957515 complexes. This indicates moderate structural stability but potentially more conformational flexibility than other systems compounds. Overall, the PCA results suggest that ligand binding particularly by GNE6776, PubChem 114917, and PubChem 162957515 contributes to the stabilization of USP conformation, with a noticeable reduction in global motions compared to the unbound protein.

### 3.12. MD Simulation Replicate of the Three Complexes

**Figure 12:**
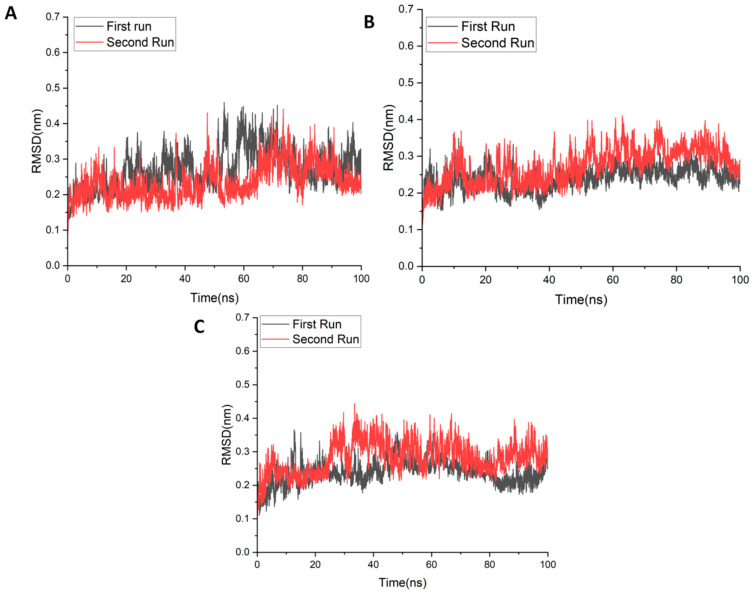
Replicate run of the MDSs for the three candidate compounds. (**A**) PubChem 162957515, (**B**) PubChem 114917, and (**C**) PubChem 442879. Black line indicates the rmsd of first MD run and red line indicates second MD run.

**Figure 13:**
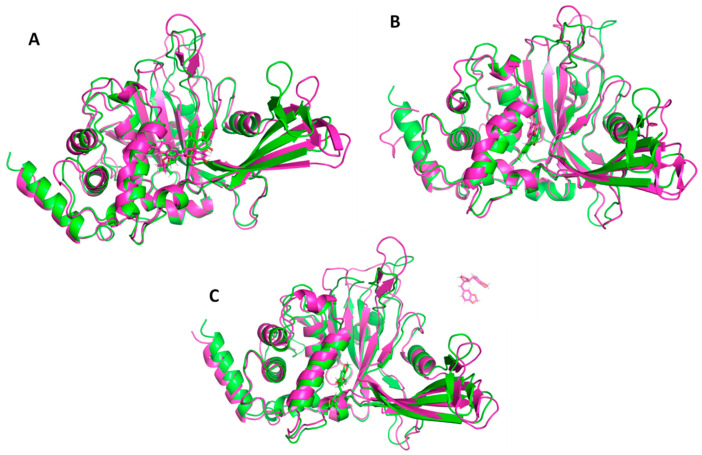
(**A**) Superimposition of the ligand–protein complexes extracted at 0 ns (green) to complex extracted at 100 ns (pink). (**A**) PubChem 162957515, (**B**) PubChem 114917, and (**C**) PubChem 442879.

To confirm the robustness and reproducibility of the molecular dynamics simulations, a second set of 100 ns MD simulations was carried out for the three top candidate compounds PubChem 162957515, PubChem 114917, and PubChem 442879. The root mean square deviation (RMSD) values of the protein backbone were compared between the first and second runs for each complex (Figure 12). Across all three candidates, the RMSD profiles from the replicate simulations showed strong consistency with their respective first runs. Minor fluctuations were observed, which are expected due to the stochastic nature of MD simulations, but the overall trends, average RMSD values, and convergence behavior were notably similar. PubChem 162957515 maintained comparable RMSD values between 0.20 and 0.40 nm in both runs, with slightly reduced fluctuation in the second run, suggesting improved conformational settling. PubChem 114917 exhibited highly overlapping RMSD curves with both runs stabilizing around ~0.25 nm, indicating reproducible structural behavior of the protein–ligand complex. PubChem 442879 demonstrated the lowest and most stable RMSD in both simulations (approximately 0.18–0.26 nm), reinforcing its potential as a highly stable binder of USP7. The high degree of similarity in RMSD trends between the two independent simulation runs affirms the reproducibility and reliability of the MD simulations. Further, the superimposition of complexes at 0 ns and 100 ns shows that, in complex PubChem 162957515 and PubChem 114917, the ligand remained stably bound within the active site throughout the simulation, indicating strong interaction stability. In contrast, in complex PubChem 442879, the ligand dissociated from the active site by the end of the simulation, suggesting weak binding affinity or structural incompatibility (Figure 13C). The same stability behavior was also observed in first run of MD simulation.

### 3.13. Molecular Mechanics/Poisson–Boltzmann Surface Area (MM-PBSA) Calculations

The binding free energy components of the simulated complexes were estimated using the MM-PBSA method (Table 7). The components include electrostatic energy, van der Waals energy, solvation free energy, gas-phase energy, and total binding free energy (Table 6). Among the complexes, reference compound GNE6776 displayed a substantial binding energy with ΔTOTAL of −25.45 kcal/mol, mostly due to favorable van der Waals and electrostatic interactions. PubChem 442879 exhibited poor binding, with a ΔTOTAL of −0.18 kcal/mol, indicating insignificant contributions from gas-phase and solvation energies. PubChem 114917 and 162957515 have considerably favorable binding energies, with ΔTOTAL values of −20.98 kcal/mol and −18.68 kcal/mol, respectively. These values indicate a strong and persistent connection. Both complexes relied heavily on favorable van der Waals and solvation energy contributions. These findings suggest that PubChem 114917 and 162957515 have strong binding affinities and are promising candidates for further investigation.

**Figure 14:**
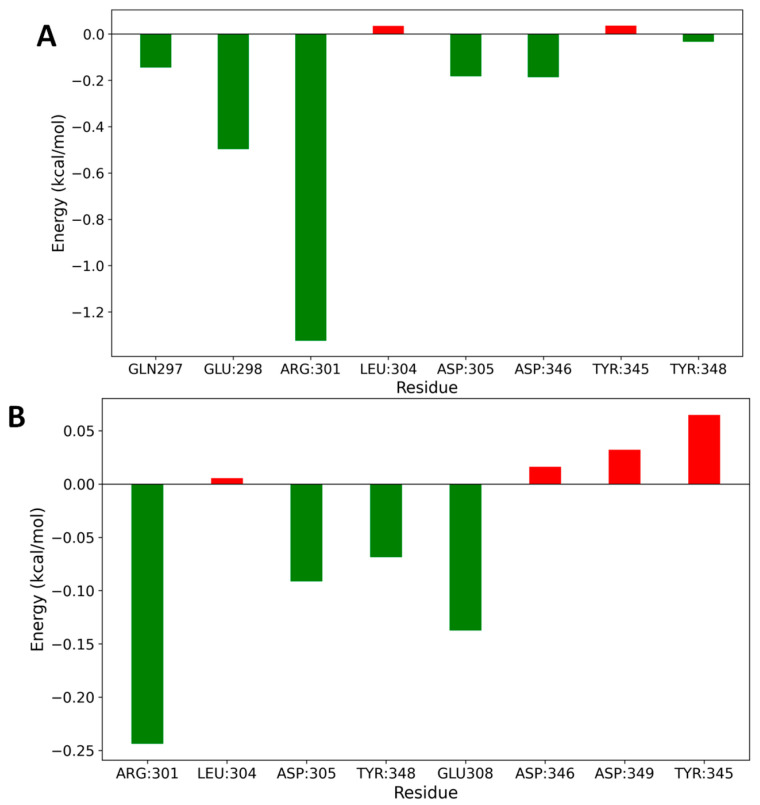
Per-residue energy decomposition analysis of the active site residues for complex (**A**) PubChem 162957515 and (**B**) PubChem 114917.

In addition, per-residue energy decomposition analysis was performed for each complex to identify the key active site residues contributing to ligand binding. This analysis provides deeper insight into the residue-level interactions that drive binding affinity and specificity, further supporting the potential of the identified compounds. The MM-PBSA per-residue decomposition for complex PubChem 114917 and 162957515 are presented in Figure 14. The energy contribution analysis reveals that in PubChem 162957515, key residues such as ARG301 (−1.324 kcal/mol) and GLU298 (−0.4971 kcal/mol) significantly stabilize the interaction, while residues like LEU304 and TYR345 show minimal or slightly positive energy contributions. In PubChem 114917 ARG301 again contributes to stability with value (−0.2437 kcal/mol), and other residues such as GLU308 and ASP305 also show minor stabilizing roles. Overall, ARG301 emerges as a significant stabilizing residue in both complexes, suggesting its important role in binding.

## 4. Discussion

USP7 (ubiquitin specific protease 7) is a deubiquitinating enzyme (DUB) that helps regulate protein stability by removing ubiquitin from substrate proteins [72]. Its therapeutic potential arises from its involvement in a wide range of diseases, including cancer, neurological disorders, and viral infections. USP7 regulates the dynamics of the p53 and MDM2 networks by deubiquitinating both p53 and its E3 ubiquitin ligase, Mdm2. USP7 has recently been found as a regulator of numerous other tumor-associated proteins, including PTEN, Claspin, and FOXO. As a result, it plays a role in apoptosis, DNA damage response, cell cycle control, and several other physiological functions. In addition to many confirmed genetic and functional studies, aberrant USP7 expression and activity have been consistently associated with a number of cancer types [73]. In order to prevent cancer, a number of small-molecule inhibitors, including GNE-6640 and GNE-6776, have been shown to control the enzymatic activity of USP7 by binding to the specific functional region inside the “palm” position of the enzyme [28].

The purpose of this study was to use a machine learning-based screening method to find possible inhibitors that target USP7. For this purpose, a machine learning model was built and trained using the reported known compounds having inhibitory effects against USP7. This trained evaluated model was then used to find the potential active compounds against USP7 by screening MPD3, a library of natural compounds. The screened compounds through the machine learning model predicted to be active were further filtered based on the drug-likeness criteria. This drug-likeness filtering criteria rule out 15 compounds for further investigation. Subsequently, in order to evaluate the binding interactions and binding affinity, molecular docking experiments were conducted using AutoDock Vina. Molecular docking analysis identified three compounds PubChem 162957515, 114917, and 442879 that interact with crucial active site residues occupying the same binding position as the native inhibitor GNE6776. All three compounds exhibited stronger docking scores than GNE6776 (−9.9 kcal/mol), with enhanced binding affinities. The identified compounds occupied the same allosteric binding pocket as the co-crystallized USP7 inhibitor GNE6776, and GNE6776 recovered key interactions with residues ARG301, ASP346, and ASP349 involved in ligand stabilization [28], suggesting a binding mechanism comparable to previously reported USP7 allosteric inhibitors. The ability of the identified compounds to target this regulatory pocket may hold translational significance, as allosteric inhibition offers potential advantages.

Similar to the reported inhibitors, the identified compounds in the present study interacted with crucial residues such as ARG301, ASP346, and ASP349, residues known to contribute significantly to ligand stabilization and inhibitory activity within the USP7 catalytic site. Several in silico studies have highlighted the importance of the allosteric binding pocket of USP7 as a promising therapeutic target for inhibitor development [74,75,76]. In particular, co-crystal structures of USP7 complexed with allosteric inhibitors such as GNE6776 (PDB ID: 5UQX) and GNE-6640 (PDB ID: 5UQV) demonstrated that these inhibitors bind to a ubiquitin-like allosteric pocket distinct from the catalytic active site.

Further in silico validation of these potential inhibitors was carried out using moleculra dynamics (MD) simulations and Molecular Mechanics/Poisson–Boltzmann Surface Area (MM-PBSA). The RMSD and RMSF analyses demonstrated that all complexes were stable. Furthermore, MM-PBSA simulations identified PubChem 114917 and 162957515 as possible USp7 inhibitors, with binding energies of −20.98 kcal/mol and −18.68 kcal/mol. PubChem 114917 (Tanshinone I) is derived from *Salvia miltiorrhiza*. The therapeutic potential of these compounds has been investigated in multiple diseases including cardiovascular system, anticancer, anti-inflammatory, and anti-neurodegenerative diseases. Huang et al. investigated the inhibitory effect of Tanshinone I against human leukemia cells by targeting Zeste Homolog 2 (EZH2). The suppression of EZH2 leads to reduced proliferation of leukemia cells both in vitro and in vivo [77]. Anticancer properties of Tanshinone I has also been widely investigated against breast cancer by modulating autophagy [78]. A recent study Wu et al. investigates the anti-inflammatory effects of Tanshinone I by targeting NLRP3 inflammasome, a key component in the inflammatory response [79]. Considering that USP7 plays an important role in tumor progression, immune regulation, and p53–MDM2 signaling pathways, the predicted interaction of Tanshinone I with USP7 may provide an additional mechanistic explanation for its previously reported pharmacological activities. Compound 442879 (Hinokinin) is a type of phenolic compound found in various plants. Its biological activities and pharmacological potential have been studies widely [80,81]. Hinokinin has been demonstrated to inhibit the proliferation of numerous cancer cell types. Notably, it promotes G2/M cell cycle arrest and improves the effects of chemotherapeutic drugs such as doxorubicin in breast cancer cells, indicating its potential as an anticancer agent [82]. Hinokinin has been shown to have neuroprotective properties for the treatment of neurodegenerative illnesses such as Alzheimer’s disease [83]. The computational results shed light on the binding mechanisms of compounds with USP7, suggesting their potential as inhibitors. The present study is primarily based on in silico approaches, which may not fully represent the complex biological environment in living systems. Although the identified compounds demonstrated favorable binding affinities and interaction profiles with the target USP protein, computational predictions alone cannot confirm their actual inhibitory activity or therapeutic efficacy. Further validation through in vitro and in vivo studies is necessary to confirm the inhibitory potential, selectivity, and safety profiles.

In addition, protein flexibility and dynamic conformational changes were only partially considered during the analysis, which may affect the accuracy of the predicted ligand–protein interactions. Nevertheless, the findings provide a valuable basis for future experimental investigations and USP-targeted drug discovery efforts.

## 5. Conclusions

This study sought to investigate therapeutics by targeting USP7 as a possible pharmacological target. An integrated strategy was used to screen a library of natural compounds against USP7, followed by MD simulations and free energy calculations Molecular Mechanics/Poisson–Boltzmann Surface Area (MM-PBSA). The machine learning model was used to prioritize and support the selection of biologically relevant compounds from the dataset, which were subsequently evaluated using molecular docking, molecular dynamics (MD) simulations, and MM-PBSA analysis. Molecular docking analysis revealed three compounds, PubChem 162957515, 114917, and 442879, as potential binders based on favorable binding energy scores and interactions with key residues within the USP7 active site. MD simulations and MM-PBSA confirmed that the USP7 inhibitor complexes are stable. MD simulations revealed that compound PubChem 114917, and 162957515 strongly stabilized the USP7 structure, resulting in low structural fluctuations and great stability throughout the simulation period. The MM-PBSA findings showed that compounds PubChem 114917 and 162957515 had a high binding affinity, indicating that they are effective USP7 inhibitors. Among the identified compounds, PubChem 114917 and 162957515 exhibited the most favorable computational profiles, including stable protein–ligand interactions and strong binding affinity. These findings suggest that the identified compounds may serve as promising starting points for the development of novel USP7 inhibitors.

While the computational results provide a solid foundation and valuable predictive insights into PubChem 114917 and 162957515’s inhibitory potential, experimental validation via in vitro and in vivo studies will be required to confirm their activity, evaluate pharmacological properties, and assess their therapeutic relevance in cancer and other USP7-associated diseases.

## Figures and Tables

**Table 1:** Names of the features calculated and their statistics.

Number	Features	Mean	Median	Standard Deviation
1	MolWT	338.36	301.77	132.32
2	Mol LogP	2.66	2.67	1.63
3	MaxPartialCharge	0.21	0.23	0.10
4	MinPartialCharge	−0.43	−0.38	0.142
5	RingCount	3.0	3.0	1.61
6	TPSA	64.53	55.84	36.24
7	NumAtoms	23.39	21.0	9.60
8	NumHeavyAtoms	23.39	21.0	9.60
9	NumAromaticRings	1.91	2.0	1.42
10	NumAliphaticRings	1.09	1.0	1.0
11	NumRotatableBonds	4.25	4.0	2.57
12	NumBonds	25.38	22.0	11.00
13	FractionSP3	0.46	0.41	0.28
14	NHOHCount	1.48	1.0	1.31
15	NOCount	4.90	4.0	2.75
16	NumHDonors	1.17	1.0	1.04
17	NumHAcceptors	4.38	4.0	2.41
18	FractionCSP3	0.46	0.41	0.28
19	NumAromaticCarbocycles	0.91	1.0	0.87
20	NumAliphaticCycles	1.09	1.0	1.03
21	NumAromaticHeterocycles	1.00	1.0	0.95
22	NumAromaticCycles	1.91	2.0	1.42
23	NumSaturatedRings	0.84	1.0	0.95
24	NumAliphaticCarbocycles	0.41	0.0	0.80
25	FpDensityMorgan1	1.22	1.20	0.21
26	Qed	0.617	0.64	0.18
27	NumValenceElectrons	124.30	112.0	48.66
28	Chi0	16.72	14.94	6.61
29	Chi3n	4.42	4.0	2.13
30	BalabanJ	1.92	1.90	0.55

**Table 2:** Performance evaluation of different machine learning models on test set.

Model	Accuracy	Sensitivity	Specificity	MCC	AUC
KNN	0.93	0.90	0.94	0.82	0.97
SVM	0.85	0.93	0.83	0.69	0.95
RF	0.96	0.92	0.97	0.90	0.99
NB	0.80	0.80	0.80	0.55	0.89

**Table 3:** Physiochemical properties of reference compound GNE6776 and selected compounds.

PubChem ID	2D Chemical Structure	Log p	Molecular Weight g/mol	H-Bond Donor	H-Bond Acceptor
Reference GNE6776	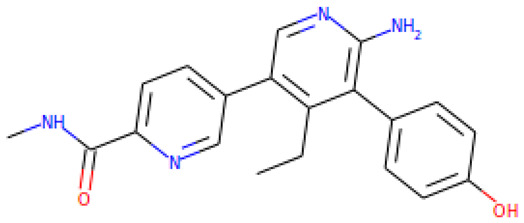	3.02	348.406	3	5
162957515	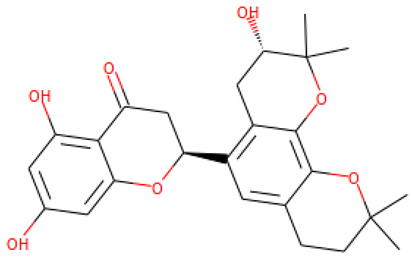	3.982	440.492	3	7
114917	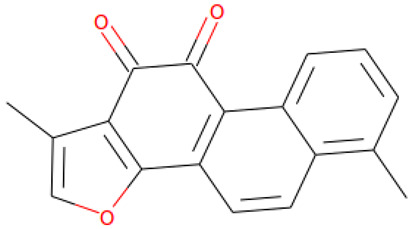	4.096	276.291	0	3
442879	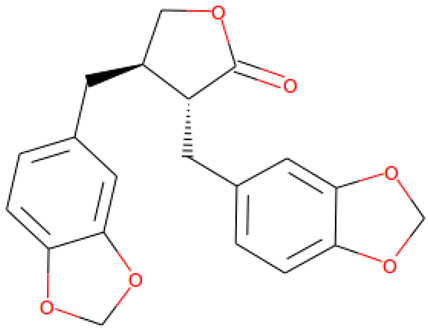	2.718	354.358	0	6

**Table 4:** Binding affinity (kcal/mol), hydrogen bonds and number of hydrogen bonds of the top selected compounds.

Protein	Compound	Binding Energy (kcal/mol)	H-Bond Interactions	Number of H-Bonds
USP7	Reference GNE6776	−9.9	Arg301, Asp305, Asp349, His403	4
	PubChem 162957515	−11.3	Glu345, Asp346, Gln297	4
	PubChem 114917	−10.6	Arg301, Asp349	3
	PubChem 442879	−10.2	Arg301, Lys312	3

**Table 5:** Biological activities of the candidate compounds predicted by PASS online server. Pa is probable activity and Pi is probable inactivity.

Compound	Activity	Pa	Pi
PubChem 442879	Anticarcinogenic	0.720	0.008
	Antineoplastic (lung cancer)	0.143	0.107
	Antineoplastic (pancreatic cancer)	0.218	0.153
	Antineoplastic (squamous cell carcinoma)	0.129	0.045
	Antineoplastic (brain cancer)	0.229	0.08
	Antineoplastic (breast cancer)	0.412	0.030
	Antineoplastic (sarcoma)	0.239	0.033
	Antineoplastic (cervical cancer)	0.20	0.04
	Antineoplastic enhancer	0.228	0.064
PubChem 114917	Antineoplastic	0.731	0.021
	Antineoplastic (breast cancer)	0.509	0.018
	Antineoplastic (cervical cancer)	0.463	0.006
	Antineoplastic (carcinoma)	0.274	0.017
	Antineoplastic (small cell lung cancer)	0.232	0.118
	Antineoplastic (ovarian cancer)	0.153	0.058
	Antineoplastic (sarcoma)	0.175	0.093
	Antineoplastic (colorectal cancer)	0.153	0.088
	Antineoplastic enhancer	0.162	0.158
PubChem 62957515	Antineoplastic	0.664	0.032
	Antineoplastic (breast cancer)	0.412	0.030
	Antineoplastic (sarcoma)	0.239	0.033
	Antineoplastic enhancer	0.203	0.064
	Antineoplastic (cervical cancer)	0.203	0.040
	Antineoplastic (brain cancer)	0.229	0.080

**Table 6:** Average values and standard deviation (SD) of RMSD, RMSF, radius of gyration (Rg) and SASA values of all the simulated systems.

Simulation System	RMSD (nm)	RMSF (nm)	Rg (nm)	SASA (nm^2^)
Apo USP7	0.23 ± 3.96	0.15 ± 1.13	2.24 ± 1.79	184.47 ± 2.71
GNE6776	0.23 ± 3.48	0.15 ± 1.12	2.26 ± 1.76	187.82 ± 3.08
PubChem 162957515	0.27 ± 5.53	0.17 ± 0.73	2.26 ± 1.66	183.83 ± 3.1
PubChem 114917	0.23 ± 2.97	0.16 ± 1.21	2.25 ± 1.34	189.32 ± 2.74
PubChem 442879	0.20 ± 3.55	0.15 ± 1.13	2.20 ± 1.92	182.44 ± 2.64

**Table 7:** Binding free energy profile (Kcal/mol) of the USP7–ligand complexes.

Complex	Kcal/mol				
	ΔEvdw	ΔEelec	ΔGGAS	ΔGSOLV	ΔTOTAL
GNE6776	−37.24 ± 2.43	−31.83 ± 4.57	−69.07 ± 4.60	43.62 ± 3.73	−25.45 ± 2.52
PubChem 442879	−0.96 ± 2.83	−0.94 ± 3.57	−1.91 ± 5.95	1.72 ± 5.24	−0.18 ± 0.97
PubChem 114917	−29.31 ± 2.61	1.61 ± 5.07	−27.70 ± 5.40	6.72 ± 4.07	−20.98 ± 3.27
PubChem 162957515	−28.60 ± 3.19	−13.61 ± 2.78	−42.21 ± 5.87	23.53 ± 7.12	−18.68 ± 4.12

## Data Availability

Data is provided within the manuscript or Supplementary Information Files.

## Supplementary Materials

The following supporting information can be downloaded at: https://www.mdpi.com/article/10.3390/cimb48060621/s1.

## References

[1] Hochstrasser M. (1996). Ubiquitin-dependent protein degradation. Annu. Rev. Genet..

[2] Hochstrasser M.J.N. (2009). Origin and function of ubiquitin-like proteins. Nature.

[3] Lee I., Schindelin H. (2008). Schindelin, Structural insights into E1-catalyzed ubiquitin activation and transfer to conjugating enzymes. Cell.

[4] Li W., Ye Y. (2008). Polyubiquitin chains: Functions, structures, and mechanisms. Cell. Mol. Life Sci..

[5] Ciechanover A., Stanhill A. (2014). The complexity of recognition of ubiquitinated substrates by the 26S proteasome. Biochim. Biophys. Acta-Mol. Cell Res..

[6] Farshi P., Deshmukh R.R., O Nwankwo J., Arkwright R.T., Cvek B., Liu J., Dou Q.P. (2015). Deubiquitinases (DUBs) and DUB inhibitors: A patent review. Expert Opin. Ther. Pat..

[7] Beck D.B., Werner A., Kastner D.L., Aksentijevich I. (2022). Disorders of ubiquitylation: Unchained inflammation. Nat. Rev. Rheumatol..

[8] Javaid S., Zadi S., Awais M., Wahab A.-T., Zafar H., Maslennikov I., Choudhary M.I. (2024). Identification of new leads against ubiquitin specific protease-7 (USP7): A step towards the potential treatment of cancers. RSC Adv..

[9] Park H.-B., Baek K.-H. (2023). Current and future directions of USP7 interactome in cancer study. Biochim. Biophys. Acta-Rev. Cancer.

[10] Nininahazwe L., Liu B., He C., Zhang H., Chen Z.-S. (2021). The emerging nature of Ubiquitin-specific protease 7 (USP7): A new target in cancer therapy. Drug Discov. Today.

[11] Valles G.J., Bezsonova I., Woodgate R., Ashton N.W. (2020). USP7 is a master regulator of genome stability. Front. Cell Dev. Biol..

[12] Bhattacharya S., Chakraborty D., Basu M., Ghosh M.K. (2018). Emerging insights into HAUSP (USP7) in physiology, cancer and other diseases. Signal Transduct. Target. Ther..

[13] He Y., Wang S., Tong J., Jiang S., Yang Y., Zhang Z., Xu Y., Zeng Y., Cao B., Moran M.F. (2020). The deubiquitinase USP7 stabilizes Maf proteins to promote myeloma cell survival. J. Biol. Chem..

[14] Zheng N., Chu M., Lin M., He Y., Wang Z. (2020). USP7 stabilizes EZH2 and enhances cancer malignant progression. Am. J. Cancer Res..

[15] Saha G., Sarkar S., Mohanta P.S., Kumar K., Chakrabarti S., Basu M., Ghosh M.K. (2022). USP7 targets XIAP for cancer progression: Establishment of a p53-independent therapeutic avenue for glioma. Oncogene.

[16] Sakamoto T., Kuboki S., Furukawa K., Takayashiki T., Takano S., Yoshizumi A., Ohtsuka M. (2023). TRIM27-USP7 complex promotes tumour progression via STAT3 activation in human hepatocellular carcinoma. Liver Int..

[17] Zhang L., Wang H., Tian L., Li H. (2016). Expression of USP7 and MARCH7 is correlated with poor prognosis in epithelial ovarian cancer. Tohoku J. Exp. Med..

[18] Sedzro D.M., Idris M.O., Durojaye O.A., Yekeen A.A., Fadahunsi A.A., Alakanse S.O. (2022). Identifying Potential p53-MDM2 Interaction Antagonists: An Integrated Approach of Pharmacophore-Based Virtual Screening, Interaction Fingerprinting, MD Simulation and DFT Studies. ChemistrySelect.

[19] Durojaye O.A., Yekeen A.A., Idris M.O., Okoro N.O., Odiba A.S., Nwanguma B.C. (2024). Investigation of the MDM2-binding potential of de novo designed peptides using enhanced sampling simulations. Int. J. Biol. Macromol..

[20] Kwon S.-K., Saindane M., Baek K.-H. (2017). p53 stability is regulated by diverse deubiquitinating enzymes. Biochim. Biophys. Acta-Rev. Cancer.

[21] Bojagora A. (2020). Identifying Novel Interactions Between Human Ubiquitin-Specific Protease 7 (USP7) and Histone Methylating/Demethylating Proteins. Master’s Thesis.

[22] Saha G., Roy S., Basu M., Ghosh M.K. (2023). USP7-a crucial regulator of cancer hallmarks. Biochim. Biophys. Acta-Rev. Cancer.

[23] Gao H., Xi Z., Dai J., Xue J., Guan X., Zhao L., Chen Z., Xing F. (2024). Drug resistance mechanisms and treatment strategies mediated by Ubiquitin-Specific Proteases (USPs) in cancers: New directions and therapeutic options. Mol. Cancer.

[24] Carreira L.D., Oliveira R.I., Moreira V.M., Salvador J.A.R. (2023). Ubiquitin-specific protease 7 (USP7): An emerging drug target for cancer treatment. Expert Opin. Ther. Targets.

[25] Pozhidaeva A. (2016). Structural Insight into the Mechanisms of Activation and Substrate Specificity of Human Deubiquitinating Enzyme USP7. Ph.D. Thesis.

[26] Khut P.-Y. (2006). Structure Function Analysis of the Deubiquitylating Enzyme Fam. Master’s Thesis.

[27] Oliveira R.I., Guedes R.A., Salvador J.A.R. (2022). Highlights in USP7 inhibitors for cancer treatment. Front. Chem..

[28] Kategaya L., Di Lello P., Rougé L., Pastor R., Clark K.R., Drummond J., Kleinheinz T., Lin E., Upton J.-P., Prakash S. (2017). USP7 small-molecule inhibitors interfere with ubiquitin binding. Nature.

[29] Dhudum R., Ganeshpurkar A., Pawar A. (2024). Revolutionizing Drug Discovery: A Comprehensive Review of AI Applications. Drugs Drug Candidates.

[30] Islam S., Hosen M.A., Ahmad S., Qamar M.T.U., Dey S., Hasan I., Fujii Y., Ozeki Y., Kawsar S.M. (2022). Synthesis, antimicrobial, anticancer activities, PASS prediction, molecular docking, molecular dynamics and pharmacokinetic studies of designed methyl α-D-glucopyranoside esters. J. Mol. Struct..

[31] Ahmed B., Ashfaq U.A., Qamar M.T.U., Ahmad M. (2014). Anticancer potential of phytochemicals against breast cancer: Molecular docking and simulation approach. Bangladesh J. Pharmacol..

[32] Muneer I., Ahmad S., Naz A., Abbasi S.W., Alblihy A., Aloliqi A.A., Alkhayl F.F.A., Alrumaihi F., Ahmad S., El Bakri Y. (2021). Discovery of novel inhibitors from medicinal plants for v-domain ig suppressor of t-cell activation. Front. Mol. Biosci..

[33] Sadaqat M., Qamar M.T.U., Masoud M.S., Ashfaq U.A., Noor F., Fatima K., Allemailem K.S., Alrumaihi F., Almatroudi A. (2023). Advanced network pharmacology study reveals multi-pathway and multi-gene regulatory molecular mechanism of Bacopa monnieri in liver cancer based on data mining, molecular modeling, and microarray data analysis. Comput. Biol. Med..

[34] Altharawi A., Ahmad S., Alamri M.A., Qamar M.T.U. (2021). Structural insight into the binding pattern and interaction mechanism of chemotherapeutic agents with Sorcin by docking and molecular dynamic simulation. Colloids Surf. B Biointerfaces.

[35] Bashir Y., Noor F., Ahmad S., Tariq M.H., Qasim M., Qamar M.T.U., Almatroudi A., Allemailem K.S., Alrumaihi F., Alshehri F.F. (2024). Integrated virtual screening and molecular dynamics simulation approaches revealed potential natural inhibitors for DNMT1 as therapeutic solution for triple negative breast cancer. J. Biomol. Struct. Dyn..

[36] Singh P., Kaur J., Singh G., Bhatti R. (2015). Triblock conjugates: Identification of a highly potent antiinflammatory agent. J. Med. Chem..

[37] Singh P., Kaur S., Kaur J., Singh G., Bhatti R. (2016). Rational design of small peptides for optimal inhibition of cyclooxygenase-2: Development of a highly effective anti-inflammatory agent. J. Med. Chem..

[38] Kaur J., Kaur B., Singh P. (2018). Rational modification of semaxanib and sunitinib for developing a tumor growth inhibitor targeting ATP binding site of tyrosine kinase. Bioorganic Med. Chem. Lett..

[39] Arti S., Kaur K., Kaur J., Ghosh T.K., Banipal T.S., Banipal P.K. (2021). Host-guest interaction of trimethoprim drug with cyclodextrins in aqueous solutions: Calorimetric, spectroscopic, volumetric and theoretical approach. J. Mol. Liq..

[40] Kaur J., Kaur S., Singh P. (2016). Rational modification of the lead molecule: Enhancement in the anticancer and dihydrofolate reductase inhibitory activity. Bioorganic Med. Chem. Lett..

[41] Noor F., Junaid M., Almalki A.H., Almaghrabi M., Ghazanfar S., Qamar M.T.U. (2024). Deep learning pipeline for accelerating virtual screening in drug discovery. Sci. Rep..

[42] Majeed A., Qamar M.T.U., Maryam A., Mirza M.U., Alhussain L., Al Otaibi S.O., Almatroudi A., Allemailem K.S., Alrumaihi F., Aloliqi A.A. (2024). Structural insights into the mechanism of resistance to bicalutamide by the clinical mutations in androgen receptor in chemo-treatment resistant prostate cancer. J. Biomol. Struct. Dyn..

[43] Aldakheel F.M., Alduraywish S.A., Dabwan K.H. (2025). Integrating machine learning driven virtual screening and molecular dynamics simulations to identify potential inhibitors targeting PARP1 against prostate cancer. Sci. Rep..

[44] Almatroudi A. (2024). Integrative machine learning, virtual screening, and molecular modeling for BacA-Targeted Anti-Biofilm drug discovery against Staphylococcal infections. Crystals.

[45] Alhassan H.H. (2025). Integrative machine learning and molecular simulation approaches identify GSK3β inhibitors for neurodegenerative disease therapy. Sci. Rep..

[46] Bento A.P., Hersey A., Félix E., Landrum G., Gaulton A., Atkinson F., Bellis L.J., De Veij M., Leach A.R. (2020). An open source chemical structure curation pipeline using RDKit. J. Cheminformatics.

[47] Mysinger M.M., Carchia M., Irwin J.J., Shoichet B.K. (2012). Directory of useful decoys, enhanced (DUD-E): Better ligands and decoys for better benchmarking. J. Med. Chem..

[48] Maćkiewicz A., Ratajczak W. (1993). Principal components analysis (PCA). Comput. Geosci..

[49] Kramer O. (2016). Machine Learning for Evolution Strategies.

[50] Huang S., Cai N., Pacheco P.P., Narandes S., Wang Y., Xu W. (2018). Applications of support vector machine (SVM) learning in cancer genomics. Cancer Genom. Proteom..

[51] Peterson L.E. (2009). K-nearest neighbor. Scholarpedia.

[52] Webb G.I., Keogh E., Miikkulainen R. (2010). Naïve Bayes. Encycl. Mach. Learn..

[53] Qi Y. (2012). Random forest for bioinformatics. Ensemble Machine Learning: Methods Applications.

[54] Mumtaz A., Ashfaq U.A., Qamar M.T.U., Anwar F., Gulzar F., Ali M.A., Saari N., Pervez M.T. (2017). MPD3: A useful medicinal plants database for drug designing. Nat. Prod. Res..

[55] Chopra B., Dhingra A.K. (2021). Natural products: A lead for drug discovery and development. Phytother. Res..

[56] Chaachouay N., Zidane L. (2024). Plant-derived natural products: A source for drug discovery and development. Drugs Drug Candidates.

[57] Karami T.K., Hailu S., Feng S., Graham R., Gukasyan H.J. (2022). Eyes on Lipinski’s rule of five: A New “rule of thumb” for physicochemical design space of ophthalmic drugs. J. Ocul. Pharmacol. Ther..

[58] Huey R., Morris G.M., Forli S. (2012). Using AutoDock 4 and AutoDock Vina with AutoDockTools: A Tutorial.

[59] Trott O., Olson A.J. (2010). AutoDock Vina: Improving the speed and accuracy of docking with a new scoring function, efficient optimization, and multithreading. J. Comput. Chem..

[60] Abraham M.J., Murtola T., Schulz R., Páll S., Smith J.C., Hess B., Lindahl E. (2015). GROMACS: High performance molecular simulations through multi-level parallelism from laptops to supercomputers. SoftwareX.

[61] Huang J., Mackerell A.D. (2013). CHARMM36 all-atom additive protein force field: Validation based on comparison to NMR data. J. Comput. Chem..

[62] Vanommeslaeghe K., MacKerell A.D. (2012). Automation of the CHARMM General Force Field (CGenFF) I: Bond perception and atom typing. J. Chem. Inf. Model..

[63] Srivastava A., Malik S., Debnath A. (2019). Heterogeneity in structure and dynamics of water near bilayers using TIP3P and TIP4P/2005 water models. Chem. Phys..

[64] Hess B., Bekker H., Berendsen H.J., Fraaije J.G. (1997). LINCS: A linear constraint solver for molecular simulations. J. Comput. Chem..

[65] Guterres H., Im W. (2020). Improving protein-ligand docking results with high-throughput molecular dynamics simulations. J. Chem. Inf. Model..

[66] Sultana T., Mou S.I., Chatterjee D., Faruk M.O., Hosen M.I. (2024). Computational exploration of SLC14A1 genetic variants through structure modeling, protein-ligand docking, and molecular dynamics simulation. Biochem. Biophys. Rep..

[67] Shahab M., Zheng G., Khan A., Wei D., Novikov A.S. (2023). Machine learning-based virtual screening and molecular simulation approaches identified novel potential inhibitors for cancer therapy. Biomedicines.

[68] Genheden S., Ryde U. (2015). The MM/PBSA and MM/GBSA methods to estimate ligand-binding affinities. Expert Opin. Drug Discov..

[69] Valdés-Tresanco M.S., Valdés-Tresanco M.E., Valiente P.A., Moreno E. (2021). gmx_MMPBSA: A new tool to perform end-state free energy calculations with GROMACS. J. Chem. Theory Comput..

[70] Ramírez D., Caballero J. (2018). Is it reliable to take the molecular docking top scoring position as the best solution without considering available structural data?. Molecules.

[71] Erickson J.A., Jalaie M., Robertson D.H., Lewis R.A., Vieth M. (2004). Lessons in molecular recognition: The effects of ligand and protein flexibility on molecular docking accuracy. J. Med. Chem..

[72] Wang Z., Kang W., You Y., Pang J., Ren H., Suo Z., Liu H., Zheng Y. (2019). USP7: Novel drug target in cancer therapy. Front. Pharmacol..

[73] Georges A., Marcon E., Greenblatt J., Frappier L. (2018). Identification and characterization of USP7 targets in cancer cells. Sci. Rep..

[74] Wang X., Liu N., Li N., Lu S., Chai Z. (2025). Mechanistic insights into the mechanism of allosteric inhibition of ubiquitin-specific protease 7 (USP7). Biomolecules.

[75] Shahab M., Waqas M., Fahira A., Zhang H., Huang Z. (2025). Structure-guided discovery of ubiquitin-specific protease 7 inhibitors through integrative quantitative structure-activity relationship modeling, docking, and molecular dynamics. Comput. Biol. Med..

[76] Ojedele O.A., Umar H.I., Baammi S., Metouekel A., Mengistie A.A., Bin Jardan Y.A., Shazly G.A., Victor O. (2024). Cheminformatics-aided discovery of potential allosteric site modulators of ubiquitin-specific protease 7. Sci. Rep..

[77] Huang Y., Yu S.-H., Zhen W.-X., Cheng T., Wang D., Lin J.-B., Wu Y.-H., Wang Y.-F., Chen Y., Shu L.-P. (2021). Tanshinone I, a new EZH2 inhibitor restricts normal and malignant hematopoiesis through upregulation of MMP9 and ABCG2. Theranostics.

[78] Naz I., Merarchi M., Ramchandani S., Khan M.R., Malik M.N., Sarwar S., Narula A.S., Ahn K.S. (2020). An overview of the anti-cancer actions of Tanshinones, derived from Salvia miltiorrhiza (Danshen). Explor. Target. Anti-Tumor Ther..

[79] Zhao J., Liu H., Hong Z., Luo W., Mu W., Hou X., Xu G., Fang Z., Ren L., Liu T. (2023). Tanshinone I specifically suppresses NLRP3 inflammasome activation by disrupting the association of NLRP3 and ASC. Mol. Med..

[80] Schmidt T.J., A Khalid S., Romanha A.J., Alves T.M., Biavatti M.W., Brun R., Da Costa F.B., De Castro S.L., Ferreira V.F., De Lacerda M.V.G. (2012). The potential of secondary metabolites from plants as drugs or leads against protozoan neglected diseases-part II. Curr. Med. Chem..

[81] Marcotullio M.C., Pelosi A., Curini M. (2014). Hinokinin, an emerging bioactive lignan. Molecules.

[82] Cunha N.L., Teixeira G.M., Martins T.D., Souza A.R., Oliveira P.F., Símaro G.V., Rezende K.C.S., Gonçalves N.d.S., Souza D.G., Tavares D.C. (2016). (−)-Hinokinin induces G2/M arrest and contributes to the antiproliferative effects of doxorubicin in breast cancer cells. Planta Medica.

[83] Tarbiat S., Unver D., Tuncay S., Isik S., Yeman K.B., Mohseni A.R. (2023). Neuroprotective effects of Cubebin and Hinokinin lignan fractions of Piper cubeba fruit in Alzheimer’s disease in vitro model. Turk. J. Biochem..

