# Supplementary material for: Screening of Natural Product-Derived USP7 Inhibitors for Cancer Therapy via Integrated Machine Learning and Molecular Simulations
Source: Curr Issues Mol Biol. 2026 Jun 16;48(6):621. doi: 10.3390/cimb48060621 (PMC13297843; doi:10.3390/cimb48060621)
Supplement: Supplementary file 1 [file cimb-48-00621-s001.zip › supplementary.pdf]

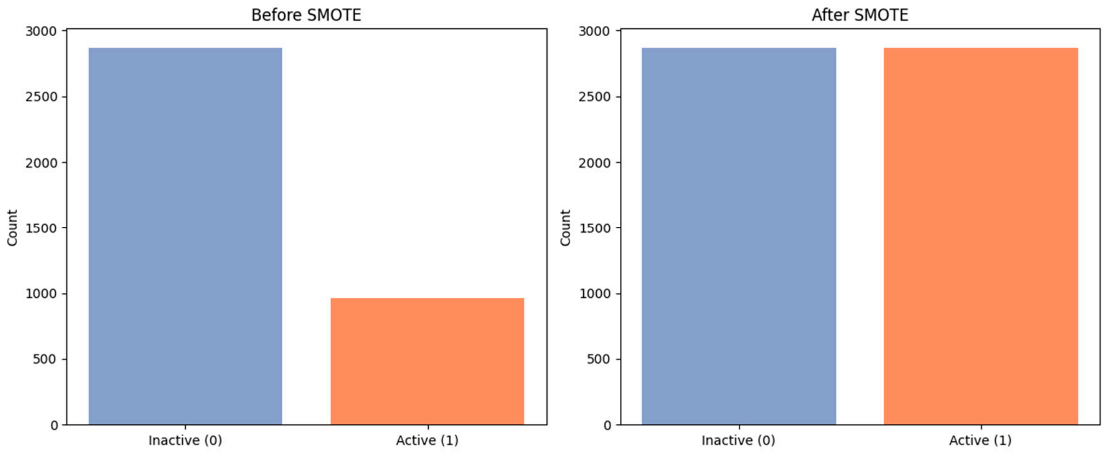

Figure S1 Class balance before and after SMOTE applied.

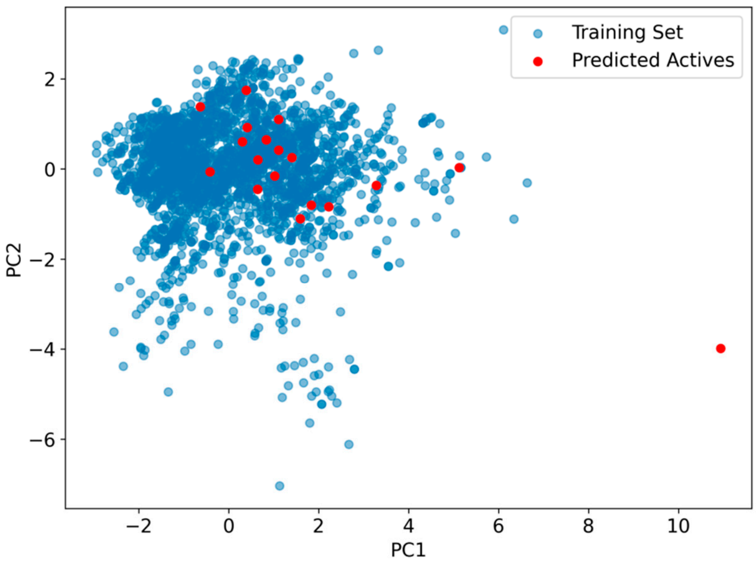

Figure S2 Applicability domain (AD) analysis of the 19 selected active compounds using PCA-based chemical space representation. Applicability domain (AD) analysis of the 19 selected active compounds using PCA-based chemical space representation.

Table S1 Docking scores (kcal/mol) of the 15 compounds resulted from the machine learning model screening.

PubChem ID	Binding Energy kcal/mol
162957515	-11.3
114917	-10.6
C442879	-10.2
10611938	-9.3
9978176	-9.3
162931346	-9.2
65126	-9.2
42607512	-8.7
160705	-8.5
15276284	-8.3
122667	-8.2
345501	-7.9
443014	-7.9
11688549	-6.7
393601	-6.2
